# Medical and Chirurgical Faculty of Maryland

**Published:** 1880-05

**Authors:** 


THE MEDICAL AND CHIRURGICAL FACULTY OF MARYLAND held its Eighty-Second Annual Meeting, April 13, 1880, in Hopkins Hall, Prof. S. C. Chew, the President, in the chair. Prof. John W.

Mallet, of the University of Virginia, delivered the annual address. His subject was "The Claims of Science for its own Sake upon the Medical Profession." A number of papers were read, and after the election of President — Dr. Wilson — and other officers, the annual meeting adjourned.

